# Incidence of Common Fusion Transcripts in Adult and Pediatric Acute Myeloid Leukemia (AML) Cases: Experience of a Tertiary Care Research institute

**DOI:** 10.4084/MJHID.2012.042

**Published:** 2012-06-20

**Authors:** Prateek Bhatia, Jogeshwar Binota, Neelam Varma, RK Marwaha, Pankaj Malhotra, Subhash Varma

**Affiliations:** 1 Assistant Professor- Pediatrics, Post Graduate Institute of Medical Education and Research, Chandigarh; 2 Senior Laboratory Technician-Hematology, Post Graduate Institute of Medical Education and Research, Chandigarh; 3 Professor and Head -Hematology, Post Graduate Institute of Medical Education and Research, Chandigarh; 4 Professor – Pediatric Hemato-oncology, Post Graduate Institute of Medical Education and Research, Chandigarh; 5 Additional Professor- Internal Medicine, Post Graduate Institute of Medical Education and Research, Chandigarh; 6 Professor & Head – Internal Medicine, Post Graduate Institute of Medical Education and Research, Chandigarh

## Abstract

**Introduction:**

The incidence of common fusion transcripts in AML is 40–45%, but data from Indian sub-continent is limited.

**Aims & Objectives:**

The aim of the present study is to note the incidence of common fusion transcripts of AML1-ETO, PML-RARA and CBFβ-MYH11 in adult and pediatric AML cases.

**Materials & Methods:**

A total of 116 AML cases diagnosed on bone marrow, cytochemistry and Flow-cytometry over a period of 2 year were enrolled and bone marrow samples in EDTA were processed by multiplex RT-PCR assay.

**Results:**

Of 116 cases, 96 (83%) were adult and 20 (17%) pediatric cases. A total of 39/116 (33.6%) cases showed positivity for fusion transcripts of which 28/96 (29.16%) were adult and 11/20 (55%) pediatric cases. Of the 28 positive adult cases, 14/96 (14.58%) were positive for AML1-ETO, 12/96 (12.5%) for PML-RARA and 2/96 (2.08%) for CBFβ-MYH11. In the 11 positive pediatric cases, 6/20 (30%) were positive for AML1-ETO, 3/20 (15%) for PML-RARA and 2/20 (10%) for CBFβ-MYH11.

**Discussion & Conclusion:**

The incidence of the common fusion transcripts in our pilot study is in accordance with that described in western studies. It is important to identify these transcripts as they provide useful prognostic information to the treating clinician.

## Introduction

The present WHO 2008 classification is based on morphology, immunophenotyping, flow cytometry, cytogenetics and molecular studies and classifies AML into different categories. AML with recurrent genetic abnormalities occupies an important sub-group as the universal criteria for 20% blasts is not necessary for diagnosis of leukemia, if any of the abnormalities in the sub-group are present. Moreover, the different genetic alterations imply prognostic information to the treating clinician regarding the disease behavior and outcome. Though most of the recurrent translocations can be detected using conventional karyotyping or Fluorescent in hybridization (FISH) technique, but these are either cumbersome or costly and depend upon good quality metaphases. Multiplex Reverse transcriptase PCR is a relatively cost effective and sensitive procedure to detect the common fusion transcripts and can be utilized as an initial diagnostic screening assay in AML cases.

Though many western studies have highlighted the incidence of common fusion transcripts in AML, the data from our sub-continent is limited. The aim of the present pilot study is to note the incidence of the common chimeric fusion transcripts of AML1-ETO, PML-RARA and CBFβ-MYH11 in adult and pediatric AML cases using the multiplex RT-PCR assay.

## Methodology

The present study is a prospective study carried out over a period of 2 years (April 2010-March 2012) in the Department of Hematology of a tertiary care and referral institute (PGIMER, Chandigarh) of Northern India. The study involved 116 cases of AML diagnosed on bone marrow examination, cytochemistry and immunophenotyping. In each case a 2–3 ml Bone marrow sample was collected for multiplex RT-PCR assay. Total RNA was extracted from the sample using the commercial kit (Qiagen Miniamp RNA blood Kit-50 Reactions) according to the manufacturer’s instructions and then reverse transcriptase reaction was performed using the cDNA synthesis kit (Fermantas). The quality of cDNA was analyzed using the primers for beta-actin housekeeping gene. The multiplex RT-PCR assay was then carried out using primers specific (see Table 1 for primer sequences & product size) for each of the above transcripts as per the protocol designed by S. Pakakasama et al^1^ in their study. In addition to the above Multiplex RT-PCR, another Multiplex RT-PCR was carried out separately in suspected AML-M3 cases to detect all the three isoforms of PML-RARA i.e. Bcr 1 (intron 6): Long isoform; Bcr 2 (exon 6): Variable isoform and Bcr 3 (intron 3): Short isoform (see Table 1 for primer sequences and PCR product sizes for the primer pairs). This PCR assay was carried out in following conditions- Pre-Denaturation- 95c-1 mt (one cycle); Denaturation- 94c- 1mt; Annealing- 65c-1 mt; Extension- 72c- 1mt; (A total of 35 cycles); No final extension needed. The PCR products were then run on agarose gel and stained with ethidium bromide and visualized under UV-Gel doc system. Positive controls for AML1-ETO and CBFβ-MYH11 were obtained from Christian Medical College (CMC) Vellore, India. For PML-RARA, bcr1, bcr2 and bcr 3 plasmids were used as positive control in each run.

## Ethical Justification

The blood sample used in the study is withdrawn as a part of routine diagnostic work-up of the patient and no additional sample pricks were performed. Prior informed consent was taken from all patients/guardians before withdrawl of sample.

## Results

**Figure 1:**
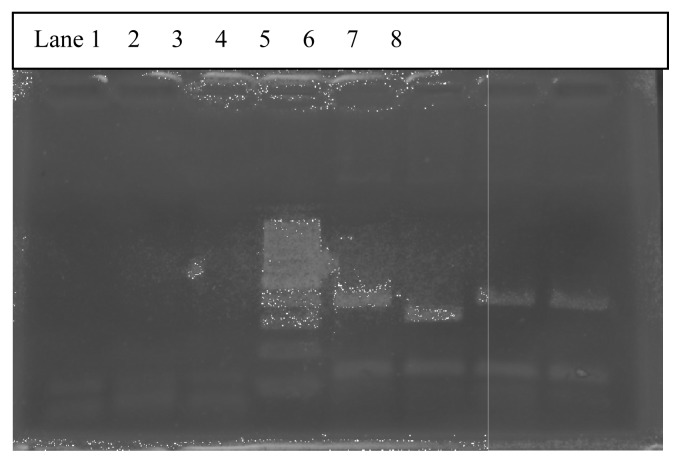
Multiplex RT-PCR gel highlighting positivity for different fusion transcripts in AML cases. Lane 3: Negative control. Lane 5: Positive for Inv16 (418bp). Lane 6: Positive for PML-RARA (381bp). Lane 7: Positive for AML1-ETO (395bp). Lane 8: Positive Control AML1-ETO (395bp)

**Figure 2:**
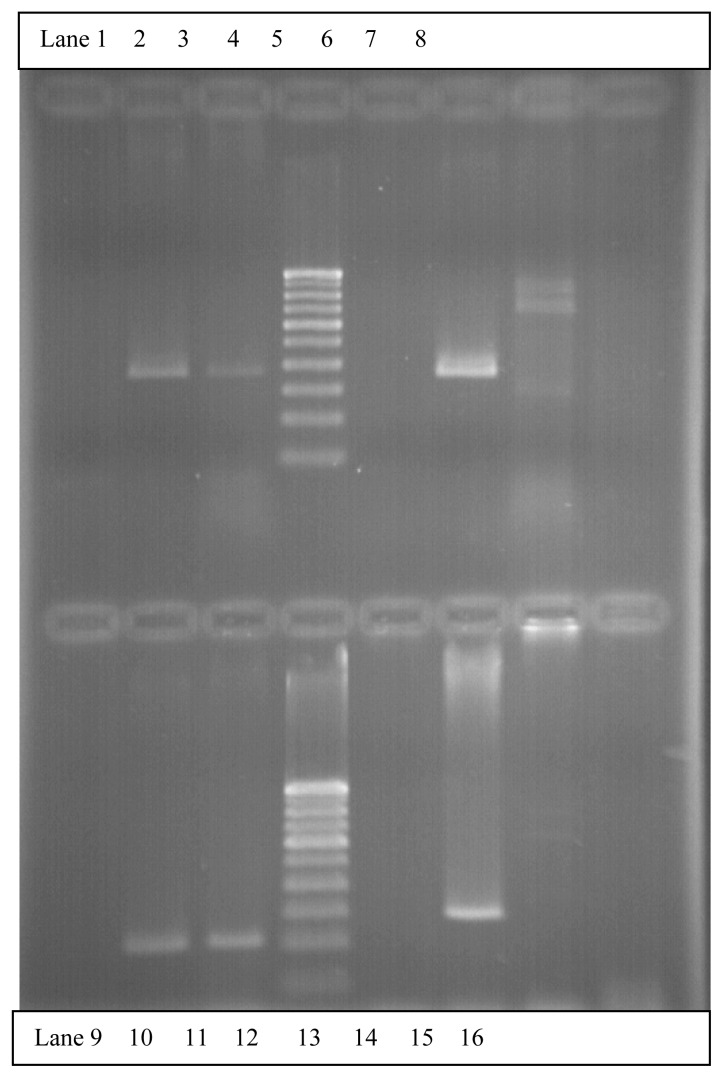
Multiplex RT-PCR gel for PML-RARA showing bcr1 isoform positivity. Lane 2: Positive control bcr1 (A1-B primer set)-381bp. Lane 3: A case of AML-M3 positive for bcr1 isoform. Lane 6: Positive control bcr3 (A2-B primer set)- 376bp. Lane 10: Positive control bcr1 (C1-D primer set)- 214bp. Lane 11: Same case of AML-M3 positive for bcr1 isoform. Lane 14: Positive control bcr3 (C2-D primer set)- 289bp. Lane 1, 5, 9, 13: Negative controls with each primer set.

The multiplex RT-PCR reactions were carried out in a total of 116 AML cases. Of these, 96 (83%) were adult and 20 (17%) pediatric cases. The median age in AML cases for adults was 42 years (range 14–40 years) and for pediatric was 6 years (range 0.6–12 years). The male to female ratio in adult AML cases was 1:1 and pediatric 2:1. A total of 39/116 (33.6%) cases showed positivity for fusion transcripts of which 28/96 (29.16%) were adult and 11/20 (55%) pediatric cases. The percentage positivity for the common fusion transcripts in adult and pediatric cases is outlined in Table 2. Figure 1 shows the positive band positions for the different chimeric transcripts in AML cases and Figure 2 highlights the positivity for bcr1 isoform of PML-RARA in the separately run multiplex RT-PCR.

## Discussion

In the present study, multiplex RT-PCR was performed for common fusion transcripts of AML1-ETO, PML-RARA and CBFβ-MYH11 as it is a very cost effective screening procedure in a resource constraint setting.

The incidence of the common fusion transcripts in present study was 33.6%. The same is in accordance with other studies from Asian^1^,^2^ and Western^3^,^4^,^5^ countries. Table 3 and 4 highlights the positivity of common fusion transcripts in various AML studies in both adult and pediatric cases.

AML1-ETO was the most common fusion transcript noted in both adult (14.58%) and pediatric (30%) cases. The incidence of AML1-ETO and PML-RARA is higher in our adult AML cases as compared to the study in west. This can be partly explained due to a smaller sample size in our study, more clustering of cases as our being a tertiary referral hospital or due to certain ethnic, genetic and environmental factors, which need to be evaluated in further prospective studies. In all 39 positive cases a retrospective evaluation of morphology was also done to do a morphological-genotypic correlation. A concordance rate of 82% was noted between morphology and genotype in adult cases and 91% in pediatric cases. The classical morphology was able to predict genotype accurately (100%) in inv16 positive cases i.e. AML-M4 with Eo on morphology. This was followed by an accuracy of 85% for AML-ETO cases (AML-M2 with Eo) and 80% for AML-M3 cases (classical promyelocytes with aeur rods). Lower predictability for AML-ETO and PML-RARA is noted because cases with classical morphology were only considered but it is well described that AML-M1 cases on morphology can also show AML-ETO positivity and PML-RARA positivity can also be seen in other morphological variants of AML-M3. However there was a better correlation with genotype and morphology in PML-RARA cases if immunophenotype findings of CD34 and HLA-DR negativity were taken into account (95%).

Disease monitoring by RT-PCR is presently being done only for PML-RARA cases in our institute, with evaluation at either post induction or post consolidation phase in cases with complete morphological and hematological remission. In present study, follow-up data on RT-PCR is available for only 4 adult and 1 pediatric case positive for PML-RARA. Of these all were negative for PML-RARA transcript by RT-PCR post consolidation except for one adult case which showed persistent positivity for bcr1 transcript of PML-RARA on RT-PCR despite hematological and morphological remission.

Various studies^6^–^8^ have highlighted comparable or slightly higher sensitivity of RT-PCR as compared to cytogenetic/FISH analysis in detecting recurrent fusion transcripts in AML. It is impressed upon in these studies that routine cytogenetic analysis should be done in all AML cases to identify genetic abnormalities in AML and RT-PCR should always supplement it to define variant/cryptic translocations or detect fusion transcripts in cases with poor quality metaphases on cytogenetics. However we could not carry out detailed cytogenetic analysis or FISH in any of our AML cases due to technical limitations and cost factor and hence a comparison could not be highlighted between these techniques in our study.

## Take Home Message

Multiplex RT-PCR is not only a sensitive technique than conventional cytogenetics or but also is easy to perform and less time consuming.

Screening for the common fusion transcripts should be routinely carried out in all AML cases as it guides the clinician in taking important therapeutic decisions and provides prognostic information.

## Figures and Tables

**Table 1:** Primer sequences used in both the multiplex RT-PCR assay’s and the product sizes of the common transcripts.

Transcript genes	Primer Sequences	Product size (bp)
AML ETO	CTACCGCAGCCATGAAGAACC AGAGGAAGGCCCATTGCTGAA	395
PML RARA	CAGTGTACGCCTTCTCCATCA GCTTGTAGATGCGGGGTAGA	381
CBFβ MYH11	GCAGGCAAGGTATATTTGAAGG TCCTCTTCTCCTCATTCTGCTC	418
**Transcript genes**	**Primer Sequences**	**Product size (bp)**
PML-A1	CAGTGTACGCCTTCTCCATCA	bcr1(A1-B)-381 bcr2 (A1-B)-345 bcr1 (A2-B)-1329 bcr2 (A2-B)- 819 bcr3 (A2-B)- 376 bcr1(C1-D)-214 bcr2 (C1-D)-178
PML-A2	CTGCTGGAGGCTGTGGAC	
RARA-B	GCTTGTAGATGCGGGGTAGA	
PML-C1	TCAAGATGGAGTCTGAGGAGG	bcr1 (C2-D)-688 bcr2 (C2-D)- 652 bcr3 (C2-D)- 289

**Table II:** Positivity of adult and pediatric cases for different fusion transcripts

Age group	Positive cases	AML1-ETO	PML-RARA	CBFB-MYH11
Adult	28/96 (29.16%)	14/96 (14.58%)	12/96 (12.5%)	2/96 (2.08%)
Pediatric	11/20 (55%)	6/20 (30%)	3/20 (15%)	2/20 (10%)

**Table 3:** Shows the incidence of common transcripts in AML in various studies

Study	Number of AML cases	Incidence of Fusion Transcripts
1) **Present study**	**116**	**39 (33.6%)**
2) Olesen et al^3^	233	54 (23%)
3) Strehl et al^4^	67	24 (44%)
4) Pakakasama et al^1^	20	5 (25%)
5) Pallisgard et al^5^	102	45 (44%)
6) Hyun-Jung Choi et al^2^	213	83(39%)

**Table 4:** Highlights positivity of various fusion transcripts in adult and pediatric studies

Study	Adult			Pediatric		
	AML-ETO	PML-RARA	CBFβ-MYH11	AML-ETO	PML-RARA	CBFβ-MYH11
1. Present study	14.58%	12.5%	2.08%	30.0%	15.0%	10.0%
2. Pakakasama et al^1^	-	-	-	20%	5%	-
3. Olesen et al^3^	4.6%	4.2%	6.6%	22.2%	22.2%	5.5%
4. Hyung-Jung Choi et al^2^	12.6%	16.3%	1.6%	30.4%	17.4%	-
